# Compressed-Sensing Reconstruction Based on Block Sparse Bayesian Learning in Bearing-Condition Monitoring

**DOI:** 10.3390/s17061454

**Published:** 2017-06-21

**Authors:** Jiedi Sun, Yang Yu, Jiangtao Wen

**Affiliations:** 1 School of Information Science and Engineering, Yanshan University, 438, Hebei Avenue, Qinhuangdao 066004, China; 2 Key Laboratory of Measurement Technology and Instrumentation of Hebei Province, Yanshan University, Qinhuangdao 066004, China; paulyu2016@163.com (Y.Y.); wens2002@163.com (J.W.)

**Keywords:** compressed sensing reconstruction, sparse Bayesian learning, block sparse structure, bearing condition monitoring, wireless sensor network

## Abstract

Remote monitoring of bearing conditions, using wireless sensor network (WSN), is a developing trend in the industrial field. In complicated industrial environments, WSN face three main constraints: low energy, less memory, and low operational capability. Conventional data-compression methods, which concentrate on data compression only, cannot overcome these limitations. Aiming at these problems, this paper proposed a compressed data acquisition and reconstruction scheme based on Compressed Sensing (CS) which is a novel signal-processing technique and applied it for bearing conditions monitoring via WSN. The compressed data acquisition is realized by projection transformation and can greatly reduce the data volume, which needs the nodes to process and transmit. The reconstruction of original signals is achieved in the host computer by complicated algorithms. The bearing vibration signals not only exhibit the sparsity property, but also have specific structures. This paper introduced the block sparse Bayesian learning (BSBL) algorithm which works by utilizing the block property and inherent structures of signals to reconstruct CS sparsity coefficients of transform domains and further recover the original signals. By using the BSBL, CS reconstruction can be improved remarkably. Experiments and analyses showed that BSBL method has good performance and is suitable for practical bearing-condition monitoring.

## 1. Introduction

As critical components in rotating machinery, bearings that are not in a good condition can cause frequent machinery breakdowns [1], and these faults may result in equipment instability, poor efficiency, and even major production-safety accidents [2]. A stable machine-condition monitoring (MCM) system is required to guarantee the optimal states of the bearings during operation [3]. Various physical properties can be utilized to monitor and diagnose the bearing faults. Most MCM systems in the industrial field are based on vibration signals, which are easy to acquire and can provide complete information [4].

In modern industries, some problems exist in online wired MCM systems, such as installation difficulty and high cost, limited power supply and additional long cables. The wireless sensor network (WSN) offers a novel approach to improve the traditional wired MCM systems [5], and it has some advantages such as rapid deployment, removability, and low energy consumption [6]. However, the WSN manifests a number of limitations when applied in vibration-based MCM systems. According to the Nyquist–Shannon sampling theorem, an analog-to-digital converter (ADC) in WSN nodes needs to work at a high frequency because of the high speeds of rolling bearings [7], so does the data processing rates. For effective condition monitoring and fault diagnosis, mass memory should be used to store large packets of vibration signals. In addition, transmitting huge amounts of data via radio-frequency modules increases the time and energy consumption [8]. Thus, reducing the data size before transmission is the best way to solve the above-mentioned problems.

Many compression methods have been introduced for the machinery vibration signals in WSN. However, a serious problem is that almost all these methods require collecting original vibration signals according to sample theorem, and then applying complex algorithms to compress data in node processers. Essentially, this processing does not reduce the workload. As an emerged theory, Compressed Sensing (CS) [9] shows great promise in compressing/reconstructing data with low energy consumption. CS framework utilizes a nonlinear projection to preserve the original information in a small quantity of measurement vectors, and then reconstruct the original signals through optimization algorithms [10]. When CS is introduced in WSN systems, signal-acquisition and data-compression can be processed simultaneously in a sensor node, while the reconstruction process is operated at remote host computers [11]. This huge advantage promotes the application of CS in vibration-based MCM systems using WSN.

For the first few years after the introduction of CS, not much attention to its application was paid in the machinery-condition monitoring field. For the past two years, a few researchers have been studying the use of CS in machinery fault diagnosis. Zhou [12] mentioned an approach based on sparse representation for fault diagnosis in induction motors. The original feature vectors that described the different faults were extracted from the training samples and kept as the reference. Then, it compared the original feature vectors and real-time features, which are extracted from real time signals to identify the fault. The features extraction was based on orthogonal sparse basis representation, and the sparse solution problem was solved by l1-minimization. Zhang [13] proposed a bearing-fault diagnosis method, which combined the CS theory with the K-SVD dictionary learning algorithm. Several over-complete dictionaries were trained using the historical data of particular operating states. Only with the over-complete dictionary that represents the same fault type with the original signal, the reconstruction signal with matching pursuit (MP) algorithm has the smallest error. The bearing fault can be identified in accordance with the differences. Wang [14] developed a framework for remote machine-condition monitoring based on CS. The faults signals were transformed to the projection space by a measurement matrix that achieved the compressed acquisition and then the compressed data was transmitted to the host computer. Fast iterative shrinkage-thresholding algorithm and parallel proximal decomposition algorithm were combined for CS reconstruction. The reconstructed signals preserve the time-frequency representation signatures of the fault signals and the condition states were diagnosed with traditional methods. Zhu [15] introduced the CS and sparse-decomposition theory for machine and process monitoring. Reference [15] focused on several state-of-the-art applications, especially, the sparse-decomposition-based fault diagnosis. The referenced fault-diagnosis methods were all based on the fact that the fault signal could be constructed using weighted linear combinations of the fault samples in the learned dictionary. The fault types can be identified by comparing the representation coefficients and the reconstruction errors. Wang [16] studied a multiple down-sampling method combined with CS for bearing-fault diagnosis. CS was used to further reduce the amount of data which had been processed by the down-sampling method. Then, the compressive data were partially reconstructed by setting a proper sparsity degree via MP algorithm. The fault features could be identified through the incomplete reconstruction signals which included the specific harmonic components. Du [17] proposed a feature-identification scheme with CS, which did not need to recover the entire original data. The fault features could be extracted directly and quickly in the compressed domain. Reference [17] introduced an alternating direction method of multipliers to solve the CS reconstruction problem.

Through the literature review, we noticed that bearings faults were diagnosed based on the features that were extracted from the entirely or partially reconstructed signals from compressed samples. Therefore, the accuracy level of recovered signal directly influenced the feature extraction and fault recognition. The aforementioned methods can identify faults that have demonstrated distinct and known fault features, especially they are effective when the bearings have a single point of failure. When the bearings have compound faults or unknown fault, however, the signals will present complicated waveforms. Under this circumstance, complete information recovery of the vibration signals is important. Therefore, a high-precision reconstruction algorithm plays a vital role. Almost all the above-mentioned methods use traditional CS reconstruction algorithms. Although these algorithms have good popularity, they are not quite suitable for bearing vibration signals, because these algorithms consider only the sparsity property of the vibration signals. In general, the bearing vibration signals not only have the sparsity property, but also have specific structural features. The structured sparsity model transcends the simple sparsity models. They reduce the size of measurements required to recover a signal steadily. On the other hand, during reconstruction, they enable the users to differentiate the useful signal from the interference in a better manner [18]. Thus, by utilizing the structured sparsity model, it is possible to outperform the state-of-the-art conventional reconstruction algorithms.

Block sparse Bayesian learning (BSBL) [19] has the potential to solve the reconstruction problem. BSBL derives from Sparse Bayesian learning (SBL) methodology [20], which was first proposed for regression and classification in machine learning and was introduced for signals with block structures. BSBL not only recovers signals with block structures but also considers the intra-block correlation. BSBL outperforms the traditional CS algorithms and has the capacity to recover non-sparse signals with high precision. It has been successfully applied in the monitoring of fetal electrocardiogram (FECG) and electroencephalogram (EEG) via wireless body-area networks [21]. However, until now, it has not attracted much attention in the field of machinery vibration signals.

Based on the properties of the bearing vibration signals and related CS theory, this paper proposed a new reconstruction method combining BSBL and sparsity in transform domain to improve the recovery accuracy. The low-dimension measurement data were acquired in the sensor nodes of WSN, transmitted to workstations via radio frequency (RF) and then recovered using proposed method in the host computer. The bearing conditions can be identified on the basis of reconstructed signals. Experimental results show the reconstruction performance is better than the conventional reconstruction algorithms.

The remainder is organized as follows: Section 2 introduces the principle of Compressed Sensing and BSBL. Section 3 details the bearing vibration signal features and commonly used transform domains. The proposed method for bearing-condition monitoring via WSN is also presented in this section. Section 4 presents the experiment results and analysis for the reconstruction methods of the bearing vibration signals. We analyze the reconstruction performances and compare the results of BSBL with other methods, and also discuss the influences of the block sizes and signal-noise ratio.

## 2. Theoretical Background

This section briefly introduces the theories: Compressed Sensing and block sparse Bayesian learning.

### 2.1. Compressed Sensing Framework

In order to inspect the health conditions of rotating machinery thoroughly, condition monitoring systems are used to collect real-time data from machineries. The monitoring system established at the foundations of traditional Nyquist sampling will collect a large amount of data over a long period of operation. Therefore, it will require massive storage space and transmission capabilities, which prevents the successful application of WSN in condition monitoring. The CS theory breaks the conventional concepts of signal acquisition. CS incorporates data acquisition and compression into one procedure by capturing a small number of samples, which contains most of valuable information [10]. The original signal in time-frequency is mapped to the projection space by a measurement matrix according to CS and the compressed data are achieved. At host computer, the compressed signal can be recovered from these few samples by a particular reconstruction algorithm [22]. The fundamental noisy pattern of CS can be formed as:

(1) y=Φx+v

where $x\in R^{N\times 1}$ is the high-dimensional vector to be sensed, $\Phi \in R^{M\times N}$ is the measurement matrix where M≪N, $y\in R^{M\times 1}$ is the low-dimensional measurement vector, and v is a noise vector modeling errors incurred during the compression process or noise in the CS system [19]. In many practical applications, hardly any signal *x* is sparse in time domain, however sparse in a transform domain, thus Equation (1) can be written as:

(2) x=Ψθ

where $\Psi \in R^{N\times N}$ is a sparse representation dictionary, which is usually an orthonormal basis matrix of a transform domain. θ is the representation coefficient vector which is sparse. If the choice of the transform basis is appropriate, most of the projection coefficients in θ will be sufficiently small. If only K coefficients are nonzero in θ, the signal is considered to be K-sparse. Equation (1) can be written as:

(3) y=ΦΨθ+v=Θθ+v

where $\Theta \in R^{M\times N}$ is the sensing matrix. In Equation (3), the locations of the largest coefficients in θ that contain important information are unknown. In addition, the measurement vector y is a low-dimensional signal and θ is a high-dimensional signal. Thus, solving θ using the measurement vector y and the sensing matrix Θ is a nonlinear optimization problem. The objective is to find the sparsest solution for θ. Once θ has been recovered, x can be reconstructed via Ψ.

Next, we introduce three types of traditional recovery algorithms in CS [22]. The first is Convex Relaxation class which solves a convex optimization problem based on linear programming. Basis pursuit (BP), BP de-noising (BPDN) [23], least absolute shrinkage and selection operator (LASSO) [24] are most popular examples. Another class consists of Greedy Iterative algorithms, which are very dependent on the sparse degree, and the most commonly used methods are matching pursuit (MP) [25] and orthogonal matching pursuit (OMP) [26]. Some derived algorithms are regularized orthogonal matching pursuit (ROMP) [27], compressive sampling matching pursuit (CoSaMP) [28] and sparsity adaptive matching pursuit (SAMP) [29]. These algorithms have low operation costs and high speeds [22]. The third group consists of the Iterative Thresholding algorithms which utilize soft or hard thresholding to restore the signal. The thresholding function depends on the number of iterations. Message passing, expander matching pursuits, sequential sparse matching pursuits and belief propagation are some examples of this kind of algorithms [22].

The above-mentioned reconstruction algorithms are based on the fact that many signals can be described as sparse or compressible [10]. In recent years, researches have been investigating other characteristics to support the better reconstruction. Generally, the presence of large coefficients implies additional structural features [29] and this phenomenon has attracted much attention, especially in many cases where the transform coefficients of signal exhibit additional structures in the form of nonzero values occurring in clusters. Such signals are perceived as block sparse signals [30]. With these block structures, it is possible to express the signal x as a concatenation of a series of blocks.

(4) $$x={\left[\underset{x^{T}[1]}{\underset{︸}{x_{1}...x_{d}}}...\underset{x^{T}[2]}{\underset{︸}{x_{d+1}...x_{2d}}}...\underset{x^{T}[m]}{\underset{︸}{x_{N-d+1}...x_{N}}}\right]}^{T}$$

$x_{\left[i\right]}$ is the *i*th block, and every block has the same length *d*. There are k nonzero blocks and k<m. The exact locations of the k blocks are uncertain. Using block-sparsity property probably improves the reconstruction performance compared with these methods which only consider the signal’s sparsity as done in the conventional methods. Several novel algorithms that achieve successes using this property have been researched, such as the model-based CoSaMP [18], joint sparsity matching pursuit (JSMP) [31], block-orthogonal matching pursuit (Block-OMP) [30], and orthogonal matching pursuit like algorithm combined with maximum a posteriori based on Boltzmann machine (BM-MAP-OMP) [32]. However, almost none of these existing algorithms consider the intra-block correlation, i.e., the amplitude correlation among the coefficients in each block. In practice, intra-block correlation exists extensively in various signals. Algorithms based on block sparse Bayesian Learning, which exploits the intra-block correlation, can further improve the reconstruction performance [19].

### 2.2. Block Sparse Bayesian Learning

BSBL is a recovery method for block-sparsity signals, which not only utilizes the block structures in the signals but also considers the intra-block correlation [19]. It needs less prior knowledge and has more excellent capabilities than conditional reconstruction algorithms. In BSBL, every block $x_{\left[i\right]}\in R^{d_{i}\times 1}$ is considered as a parameterized prior model based on the multivariate Gaussian distribution:

(5) $$p(x_{i};\gamma_{i},B_{i})\sim N(0,\gamma_{i}B_{i}),\;i=1,2,...,m$$

where $\gamma_{i}$ and $B_{i}$ are unknown variables. $\gamma_{i}$ is a positive parameter, which controls the block-sparsity property in x. If $\gamma_{i}=0$, the *i*th block will be zero. The sparsity among blocks can be ensured if most $\gamma_{i}$ are close to zero. The correlation structure in a block interior is perceived via $B_{i}\in R^{d_{i}\times d_{i}}$, which is a positive definite matrix. If we assume the blocks to be irrelevant, the prior probability distribution of x becomes $p\left(x_{i};\left\{\gamma_{i},B_{i}\right\}\right)\sim N\left(0,\Sigma_{0}\right)$, where $\Sigma_{0}=\mathrm{diag}\left\{\gamma_{1}B_{1},\;\gamma_{2}B_{2},\;\ldots ,\;\gamma_{m}B_{m}\right\}$. The noise vector also has a prior Gaussian distribution p(v;λ)~N(0,λI), where λ is a positive parameter. Then, the posterior probability of x can be obtained from

(6) $$p(x|y;\lambda ,{\left\{\gamma_{i}B_{i}\right\}}_{i=1}^{m})=N(\mu_{x},\Sigma_{x})$$

The two critical variables in Equation (6) are unknown, but they can be obtained from

(7) $$\mu_{x}=\Sigma_{0}\Phi^{T}{\left(\lambda I+\Phi \Sigma_{0}\Phi^{T}\right)}^{-1}y$$

and

(8) $$\Sigma_{x}={\left(\Sigma_{0}{}^{-1}+\frac{1}{\lambda }\Phi^{T}\Phi \right)}^{-1}$$

The two above-mentioned equations involve the parameters λ and ${\left\{\gamma_{i},B_{i}\right\}}_{i=1}^{m}$. If they are solved, the Maximum-A-Posteriori (MAP) value of x can be expressed by Equation (7). We can estimate these parameters through a Type-II maximum likelihood procedure. This procedure can be solved to minimize the next cost function:

(9) $$L(\Omega )=\log\left|\lambda I+\Phi \Sigma_{0}\Phi^{T}\right|+y^{T}{\left(\lambda I+\Phi \Sigma_{0}\Phi^{T}\right)}^{-1}y$$

In Equation (9), Ω consists of all unknown parameters, and can be expressed as $\Omega ≜\left\{\lambda ,\;{\left\{\gamma_{i},B_{i}\right\}}_{i=1}^{m}\right\}$. BSBL comprises three learning rules and the most important learning rule is the estimation of $\gamma_{i}$, which determines the algorithm convergence speed and recovery quality. In addition, the learning rule for λ is also important and the algorithm performance will not be good if λ is inapposite, even though $\gamma_{i}$ can be perfectly estimated. If the data are noiseless, the global minimum of Equation (9) is often the true sparse solution, which is not subject to $B_{i}$. In effect, $B_{i}$ just influences the local convergence. One can set different constraints for $B_{i}$ to prevent overfitting [19].

We can utilize Expectation Maximization (EM) to estimate λ, $\gamma_{i}$ and $B_{i}$. Consequently, a practical algorithm BSBL-EM is derived from BSBL and EM. The learning rules of BSBL-EM are detailed below. The learning rule for $\gamma_{i}$ is

(10) $$\gamma_{i}\leftarrow \frac{1}{d_{i}}Tr\left[B_{i}^{-1}\left(\Sigma_{x}^{i}+\mu_{x}^{i}{\left(\mu_{x}^{i}\right)}^{T}\right)\right]$$

where $\Sigma_{x}^{i}\in R^{d_{i}\times d_{i}}$ is the *i*th main diagonal block in $\Sigma_{x}$, and $\mu_{x}^{i}\in R^{d_{i}\times d_{i}}$ is the *i*th block in $\mu_{x}$.

The learning rule for λ is

(11) $$\lambda \leftarrow \frac{{\left\|y-\Phi \mu_{x}\right\|}_{2}^{2}+\sum_{i=1}^{m}Tr(\Sigma_{x}^{i}{\left(\Phi^{i}\right)}^{T}\Phi^{i})}{M}$$

When the signal has the same length, we set $B_{i}=B\left(\forall i\right)$ to prevent overfitting.

The learning rule for B is

(12) $$B\leftarrow \frac{1}{m}\sum_{i=1}^{m}\frac{\Sigma_{x}^{i}+\mu_{x}^{i}{\left(\mu_{x}^{i}\right)}^{T}}{\gamma_{i}}$$

BSBL-EM assumes that the signal can be divided into a series of non-overlapping blocks. Hence, the block partitions of the original signal need to be defined in BSBL-EM. However, during reconstruction, the defined block partitions need not be the same as the true block partitions. Even though the original data have no obvious block structure, the BSBL-EM is efficient [21].

## 3. Proposed Monitoring Method Based on Bearing Signal Reconstruction

This paper proposes a reconstruction approach applied in a WSN-based bearing-condition monitoring based on BSBL-EM. It fully utilizes the block sparsity property of fault vibration signals in the transform domain and achieves a better reconstruction performance compared with traditional methods. This section briefly introduces the features and the block structure of bearing vibration signal and then presents the proposed bearing-vibration signal reconstruction method.

### 3.1. Features of Bearing Vibration Signals in Time Domain and Transformation Domains

Studies show the bearing vibration signals are typical nonstationary signals and the sparsity in time-domain is not distinct [33,34]. The sparsity of the raw signal in a specific transform domain is critical in CS. The most widely used transform analysis of bearing fault is Fourier Transformation, i.e., the frequency spectra of the vibration signals. The other commonly used transform domains are discrete cosine transform (DCT) and wavelet packet transform (WPT). Taking the bearing vibration signals provided by Case Western Reserve University [35], we mainly discuss them in the transform domains and analyze the coefficients property in this paper.

**Figure 1:**
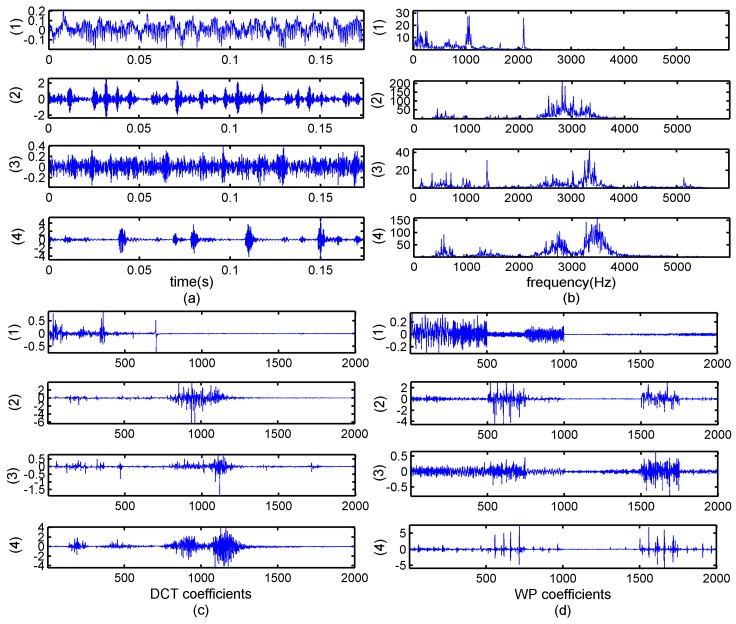
Bearing vibration signals in time domain and transform domains: (**a**) time domain; (**b**) frequency spectra; (**c**) discrete cosine transform coefficients; and (**d**) wavelet packet transform coefficients. The states in the four subpictures are: (1) normal; (2) inner ring fault; (3) rolling element fault; and (4) outer ring fault.

The data applied in this paper are the vibration signals of a deep-groove ball bearing. The specific bearing type is 6205-2RS JEM SKF. The data are collected by accelerometers, which are placed at the drive ends of the motor housing. The speed of the motor is 1750 rpm and sampling rate is 12,000 dots per second. The four states are: normal, inner-ring fault, rolling-element fault, and outer-ring fault. We used a piece of data with 2000 points as example in order to clarify the results. Figure 1 displays the original bearing vibration signals in four states and the transformation coefficients, respectively.

It can be seen from Figure 1 that the fault signals are not apparently sparse, while in Figure 1b the number of large coefficients represented major information is less than the number of small coefficients. Therefore, the bearing-fault vibration signals are much sparser in the frequency domain than in time domain. Moreover, it is obvious that the coefficients in frequency domain exhibit partition characteristics, and it can be considered that the bearing signals possess block structures. A large portion of the blocks in the mid-frequency bands and a few blocks in the other positions are nonzero. Other parts can be considered zero blocks with noise. Consequently, the original signal can be modeled as a block-sparsity signal with unknown block positions and unknown noise.

Theoretical and real signal analysis shows that the bearing vibration signals have better sparsity in transform domains than in time domain. In Figure 1c,d, we can see the coefficients of DCT and WPT showed much sparer than those of frequency domain and the block property in DCT and WPT is clearly. It is noticed that the WPT coefficients only show good sparsity and block with some fault state, for example the outer-ring fault, while the coefficients of DCT present generally spare for different fault states. Moreover DCT has the advantage of simple calculation because of the real numbers in DCT matrix and it is more beneficial to CS reconstruction algorithms.

### 3.2. Proposed Method Based on BSBL and Bearing Signal Features

**Figure 2:**
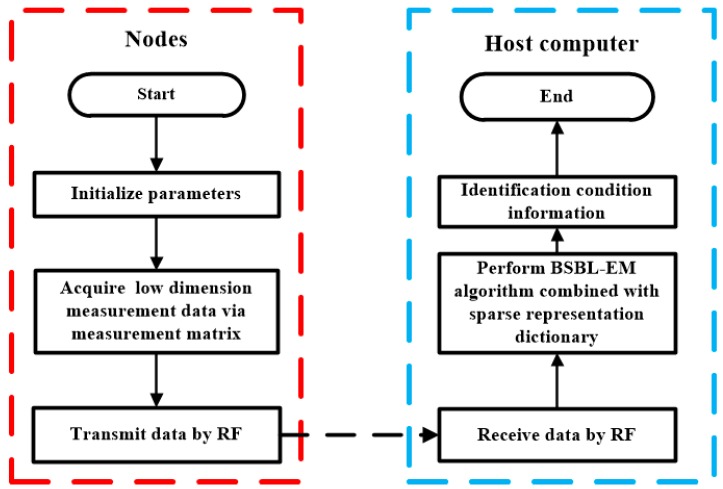
Flowchart of a machinery condition monitoring system based on wireless sensor network.

A machine-condition monitoring system based on WSN requires reducing the amount of data as much as possible before transmission. Unfortunately, most conventional data compression techniques dissipate much energy and are not suitable for the WSN. When CS is used in WSN-based conditions monitoring system, the compression stage is completed on data acquisition module before transmission, while the reconstruction stage is completed on workstations/computers at remote terminals. Then, the fault recognition about the bearing will be made according to the reconstructed signals on the host computer. Therefore, high-precision reconstruction of the original signal is essential. The bearing-condition monitoring method based on CS perfectly meets the above requirements. The flowchart of the method proposed in this paper is presented in Figure 2.

When the collection node is activated, a few initialization parameters such as measurement matrix and compression ratio will be set firstly and the sensor nodes start the vibration-data acquisition. The low-dimension measurement data can be collected via the measurement matrix. When a data-collection period is over, these temporary data are saved in memory. Next, the communication starts and the measured data are transmitted via RF modules. The host computer receives the compressed data and the BSBL-EM algorithm is performed to recover the original signals. In addition, the preset sparse representation matrix is necessary when the BSBL-EM algorithm works. Finally, the fault diagnosis is performed on the basis of high-accuracy reconstructed signal.

The procedure of proposed method is summarized:

(1) Construct the measurement matrix and choose the compression ratio.
(2) Collect the measurement signal with measurement matrix.
(3) Transmit the measurement signal to host via RF modules.
(4) Perform the BSBL-EM algorithm combined with preset sparse representation dictionary to reconstruct the signal in transformation domain.
(5) Apply the inverse transformation to get reconstruction signal in time domain.
(6) Extract the features from reconstructed signal, and apply diagnosis methods to judge fault types.

The bearing-condition monitoring method proposed in this paper combines the advantages of CS and BSBL. In the next section, we provided the experimental results based on practical bearing-vibration signals and analyzed the impacts of key factors.

## 4. Experiments and Analysis

Experiments were carried out using the bearing vibration signals provided by Case Western Reserve University. A series of experiments were performed to verify the performance of the reconstruction methods and the BSBL-EM was compared with some typical recovery algorithms. This paper also analyzed how the performance of the BSBL-EM is affected by various parameters.

### 4.1. Comparison with Traditional Reconstruction Algorithms

**Figure 3:**
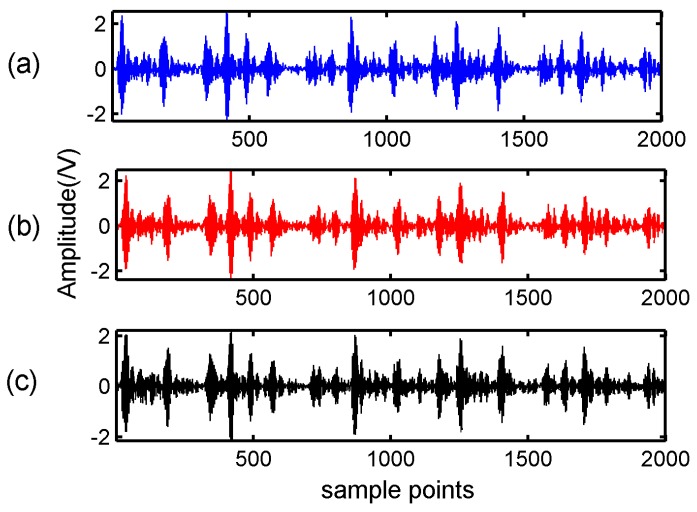
Original signal and block sparse Bayesian learning combined with expectation maximization (BSBL-EM) algorithm recovery signals: (**a**) original signal; (**b**) BSBL-EM recovery signal with discrete cosine transform; and (**c**) BSBL-EM recovery signal with wavelet packet transform.

Firstly, the BSBL-EM was verified the reconstructed performance in DCT and WPT domains. In the experiment, a bearing-vibration signal consisting of 2000 samples was chosen and in the condition of inner-ring fault. According to the features of bearing vibration signals, we chose sym6 as the wavelet packet base function. A Gaussian random matrix of size 800 × 2000 was used as the measurement matrix. The observation data can be obtained according to Equation (3). The block size of BSBL-EM in this experiment was set as 25. Figure 3 showed the original signal and the signal recovered by BSBL-EM with DCT and WPT.

**Figure 4:**
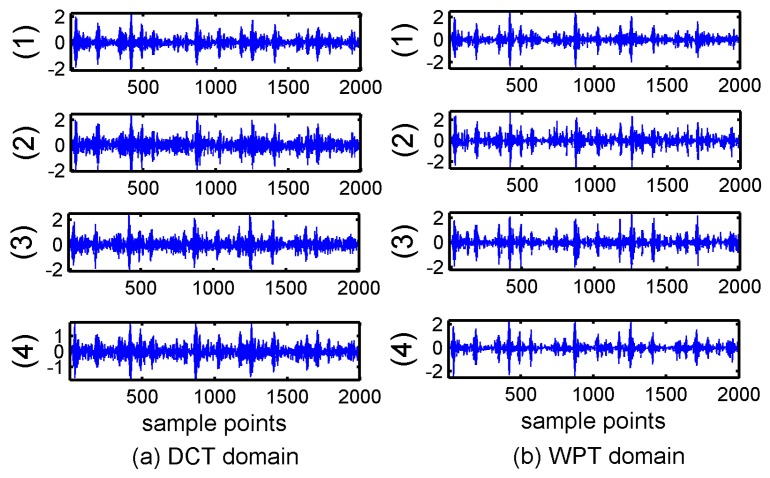
Reconstructed inner ring signals with traditional algorithms in four subpictures are: (1) Basis pursuit; (2) orthogonal matching pursuit; (3) Iterative Soft Thresholding; and (4) least absolute shrinkage and selection operator. (**a**) reconstruction signals in discrete cosine transform domain; (**b**) reconstruction signals in wavelet packet transform domain.

Secondly, BSBL-EM was compared with four classical reconstruction algorithms: BP (Basis Pursuit) [9], OMP (Orthogonal matching pursuit) [26], IST (Iterative Soft Thresholding) [36], and LASSO (Least Absolute Shrinkage and Selection Operator) [37] in DCT and WPT domains. These algorithms do not exploit the block structures of the signals. Another common feature in these four algorithms is that they do not need to know the sparsity degree or other prior information. Figure 4 showed the reconstruction results of the four algorithms.

It can be seen in Figure 3 and Figure 4 that the reconstruction signals with traditional algorithms in DCT domain and WPT domain had larger differences. The signals recovered using the traditional algorithms in DCT domain contained large amounts of noise and the signals recovered using BSBL-EM have less noise. Moreover, the reconstruction signals with BSBL-EM in two domains had similar precision. Thus, the use of the block-sparsity property to recover the original signal is an obvious advantage of the BSBL framework.

In the conditioning monitoring system based on Compressed Sensing, the compression ratio is an important factor to the performance of reconstruction signals. In Equation (1), the measurement matrix $\Phi \in R^{M\times N}$ (M≪N) fulfilled the compressive sampling in the projection space. The compression ratio (CR) can be defined as:

(13) $$\mathrm{CR}=\frac{N-M}{N}\times 100\%$$

where N is the length of the original signal and N is the length of the compressed signal.

**Figure 5:**
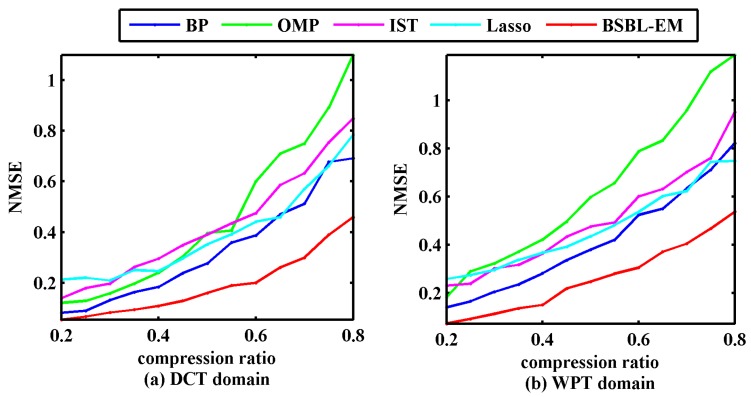
Normalized mean square errors (NMSEs) of reconstructed and original signals using five algorithms. (**a**) NMSEs in discrete cosine transform domain; (**b**) NMSEs in wavelet packet transform domain.

To further evaluate the performance of the BSBL, we experimented with the four traditional algorithms and the BSBL-EM at different compression ratios, i.e., 20–80%. The normalized mean square error (NMSE) was used as the evaluation criterion for reconstruction performance. The expression of NMSE is Equation (14).

(14) $$NMSE=\frac{{\left\|y-x\right\|}_{2}^{2}}{{\left\|x\right\|}_{2}^{2}}$$

where y is reconstruction signal and x is the original signal. Figure 5 displays the NMSEs between the original signals and the signals reconstructed via different algorithms.

**Figure 6:**
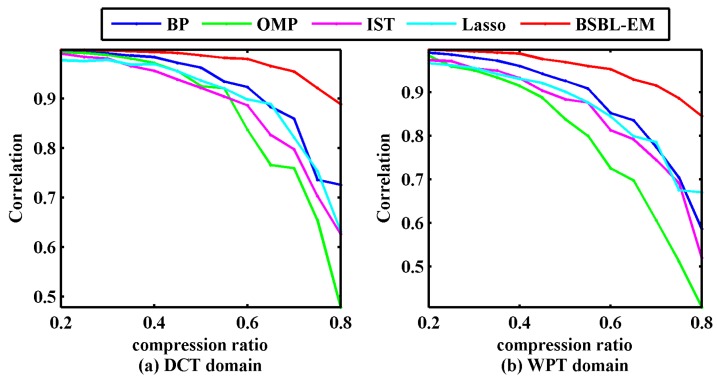
Correlation coefficients of reconstructed signals and original signals using five algorithms. (**a**) correlation coefficients in discrete cosine transform domain; (**b**) correlation coefficients in wavelet packet transform domain.

It can be seen that the reconstructed signals with BSBL-EM had smaller errors compared with the other algorithms in the given range of compression ratio. Although the NMSE can describe the reconstruction errors, NMSEs have no ability to display the similarities between original signal and the reconstructed signal. We used the Pearson correlation coefficient as Equation (14) to evaluate the similarity between the reconstructed signals and the raw signals.

(15) $$r_{xy}=\frac{n\sum xy-\sum x\sum y}{\sqrt{n\sum x^{2}-{\left(\sum x\right)}^{2}}\sqrt{n\sum y^{2}-{\left(\sum y\right)}^{2}}}$$

where $r_{xy}$ is the Pearson correlation coefficient, and x and y are the reconstructed and raw signals, respectively. Figure 6 displays the correlation coefficients between the original signals and the signals reconstructed via different algorithms under the given compression rates.

In Figure 6, the experimental results indicated that the correlation coefficient between the reconstructed signal using BSBL-EM and the original signal was above 90% when the compressibility was under 70%, irrespective of whether the sparsity representation method was DCT or WPT. This implied that BSBL-EM can recover the bearing vibration signals with satisfactory quality, ensuring subsequent fault diagnosis with high fidelity.

### 4.2. Comparison with Other Reconstruction Algorithms Utilizing Block-Sparsity Property

In Section 4.1, BSBL-EM was compared with four traditional CS algorithms which did not utilize the block-sparsity property of the bearing vibration signal. In this section, we compared the BSBL-EM with some algorithms that are based on the block structure of the original signal.

**Figure 7:**
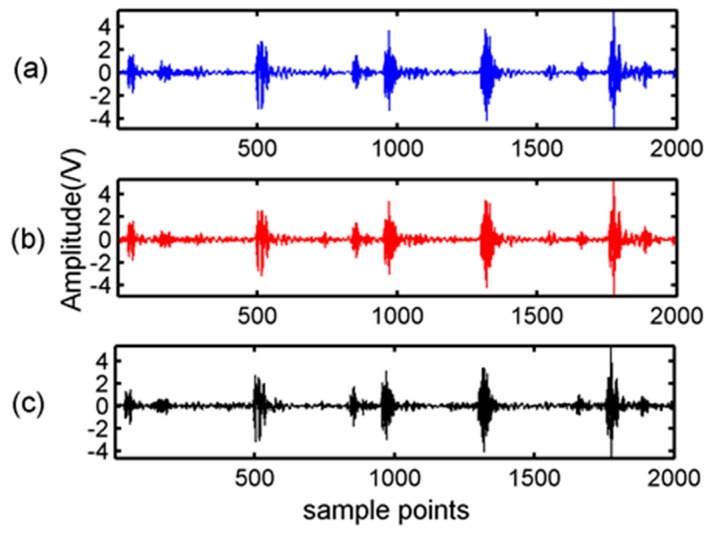
Outer fault signal and BSBL-EM recovery signals: (**a**) original signal; (**b**) BSBL-EM recovery signal with discrete cosine transform; and (**c**) BSBL-BO recovery signal with wavelet packet transform.

Sparsity representations dictionaries were DCT matrix and WPT matrix in this experiment as well. A segment of the bearing vibration signal, consisting of 2000 samples, was chosen for the experiment, and this signal was in the condition of the outer-ring fault. Other parameters were set to be the same as those used in Section 4.1. Figure 7 shows the original signal and the signal recovered using BSBL-EM.

**Figure 8:**
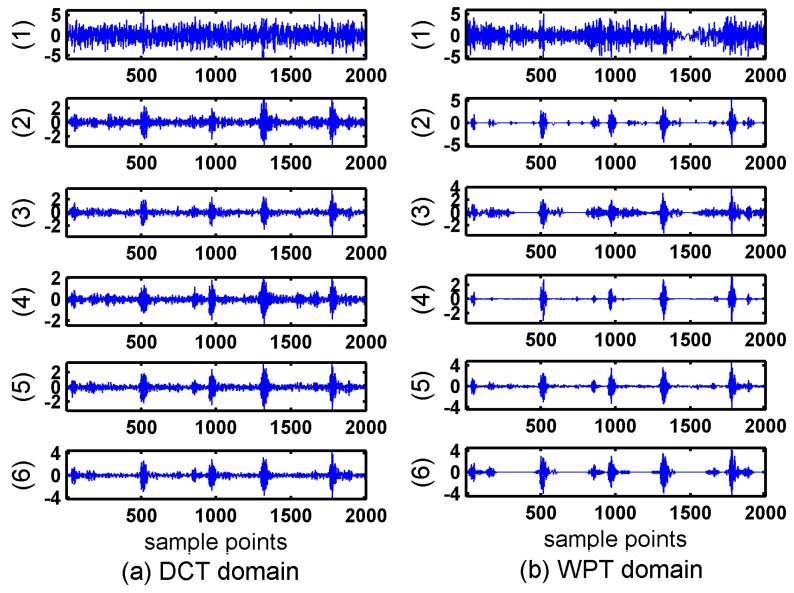
Reconstructed signals with block structure algorithms and BSBL-EM. Compared algorithms are: (1) Block orthogonal matching pursuit; (2) orthogonal matching pursuit like algorithm combined with maximum a posteriori based on Boltzmann machine; (3) joint sparsity matching pursuit; (4) Group least absolute shrinkage and selection operator; (5) Groupbasis pursuit; and (6) Struct orthogonal matching pursuit. (**a**) reconstruction signals in discrete cosine transform domain; (**b**) reconstruction signals in wavelet packet transform domain.

The compared algorithms were Block-OMP [30], BM-MAP-OMP [32], JSMP [31], Group Lasso [38], Group BP [39], and StructOMP [40]. All these algorithms exploit the block structures of the signals. A common feature of all these compared algorithms is that they all need to know the prior information. Figure 8 shows the reconstruction results of the compared algorithms with DCT and WPT.

**Figure 9:**
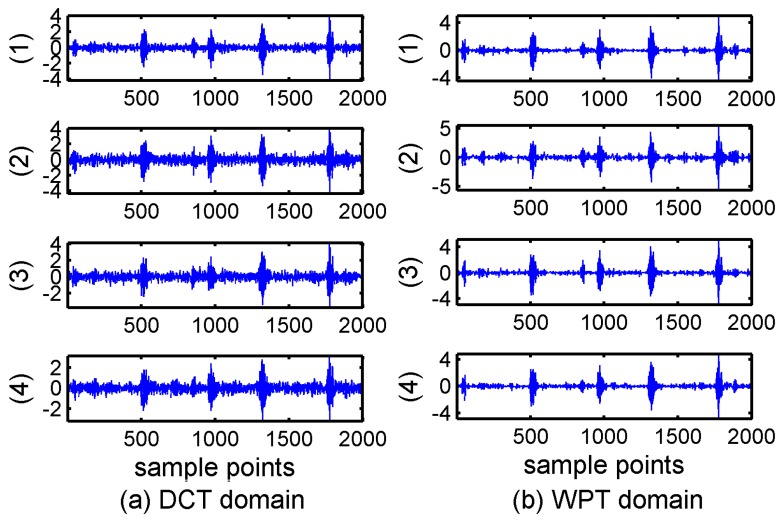
Reconstructed outer ring fault signals with traditional algorithms. Compared algorithms in four subpictures are: (1) Basis pursuit; (2) orthogonal matching pursuit; (3) Iterative Soft Thresholding; and (4) least absolute shrinkage and selection operator. (**a**) reconstruction signals in discrete cosine transform domain; (**b**) reconstruction signals in wavelet packet transform domain.

It can be seen in Figure 8 that the additional noise was serious when using the DCT. Although the noise is less when using WPT, most detail coefficients in WPT domain cannot be reconstructed successfully and the distortion in the reconstructed signals were obvious. The signals recovered via the Block OMP algorithm lost all the original information. In order to evaluate the quality of reconstruction via these block-sparsity algorithms, the outer-ring fault signal was also processed using the traditional algorithms mentioned in Section 4.1. Figure 9 shows the results of traditional algorithms in DCT and WPT domains.

Figure 8 and Figure 9 display an unexpected phenomenon. In general, the algorithms that utilize the structural properties should demonstrate better performances. However, except for the BSBL-EM, none of the algorithms using the block-sparsity property showed a precision higher than that of the traditional algorithms, in particular, the signals recovered in the wavelet packet domain. This problem can be analyzed from three angles. Firstly, it has been mentioned that the block-sparsity reconstruction methods need to know some prior information, such as the block position and size, which will directly affect the recovery results. However, the block sparse structures are actually obscure even though the energy clusters at some positions. There are also vast numbers of small coefficients at other positions, regardless of the DCT or WPT domain. Most algorithms that use the block sparse structure have been experimented under ideal conditions that have less noise, in the past researches. However, practical applications are different from numerical simulations. The structural properties of the original signal are often implicit and noise is inevitable. Secondly, these block-sparsity algorithms require corresponding structural features matched with the specific signals. However, the characteristics of the bearing vibration signals in DCT and WPT domain have great differences, as displayed in Figure 1c,d. Finally, the bearing vibration signals are typical non-stationary signals; therefore, the sparsity appears divergent in different transform domains, for example in this paper the coefficients in WPT domain showed better sparsity and block partitions than DCT domain, therefore the reconstructed results under most circumstances had better performances. Most algorithms that utilize block structures cannot achieve good reconstructed performances for the complex real bearing signals if it lacks the prior information about the block structures. In other words, excessive dependence on prior information results in the algorithms using the block-sparsity property demonstrating worse adaptability than the traditional algorithms in practical applications

BSBL framework is different from most of the algorithms that utilize the block-sparsity property. As we can see in Figure 7, the signals recovered by the BSBL-EM were almost the same whatever in DCT domain or WPT domain. BSBL-EM can well reconstruct the signal when the coefficients have inconspicuous blocks in transformation domain, even if the coefficients are non-stationary. Moreover, this algorithm only needs a minimum number of prior knowledge, namely the signals characterizing sparsity and block partitions, so the application of BSBL is as simple as that of the traditional CS algorithms.

### 4.3. Effect of Block Sizes in BSBL-EM

The improved performance of BSBL-EM over most of the typical algorithms had been displayed in the past two comparison experiments. Except the sparsity, block structure is the only prior condition needed when reconstructing the signal with BSBL-EM. In this section, we investigated the effects of different block sizes in BSBL-EM and showed the results.

**Figure 10:**
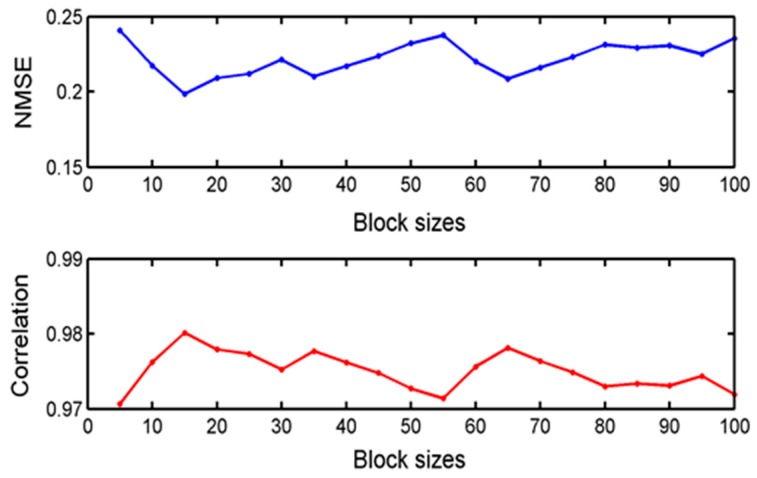
NMSE and correlation coefficients of reconstructed signals and original signal with different block sizes.

In the previous experiments, the size of each block was 25. For the block-sparsity algorithm, the block size is a key factor, so how does it affect the performance of BSBL-EM? To examine this, we used various block sizes to complete reconstruction, and the reconstructed results were satisfactory for all conditions. The raw data in this experiment are the inner-ring fault state and the measurement matrix is a Gaussian random matrix of size 800×2000 and the sparse representation dictionary is a DCT matrix. The criteria for evaluating the reconstruction performance were NMSE and correlation coefficient. The block sizes ranged from 5 to 100. Figure 10 displays the reconstructed performances of BSBL-EM for various block partitions.

According to the two evaluation criteria, the differences between the various reconstructed signals were very small. Therefore, we can consider that the performance of BSBL-EM is not sensitive to the block partitions and the effects of block partitions can be ignored.

### 4.4. Effect of SNR

In most industrial fields, the bearing vibration signals contain different levels of noise. Most often, denoising processes for acquiring data are necessary for the subsequent fault diagnosis. Therefore, it is natural to question whether the BSBL-EM can reconstruct the original signal with less noise, while at the same time, preserve important information.

**Figure 11:**
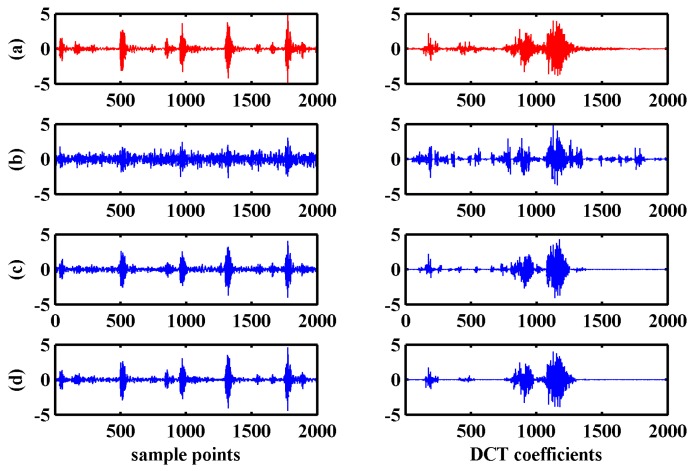
Original and the reconstructed signals and discrete cosine transform coefficients under three signal-to-noise ratios (SNRs): (**a**) original signal; (**b**) SNR = 1 dB; (**c**) SNR = 10 dB; and (**d**) SNR = 20 dB. (**a**) sample points; (**b**) discrete cosine transform coefficients.

We studied the effects of signal-to-noise ratio (SNR) and BSBL-EM will estimate noise model parameter λ according to the learning rule in Equation (11). This section explores the reconstruction effects under various noise levels. The original data were bearing outer-ring fault signals added white Gaussian noise with different SNR, measurement matrix was the same Gaussian random matrix as the previous experiment and the sparse representation dictionary was DCT matrix. Figure 11 compares the original signal and DCT coefficients with the reconstructed ones under different noise levels.

**Figure 12:**
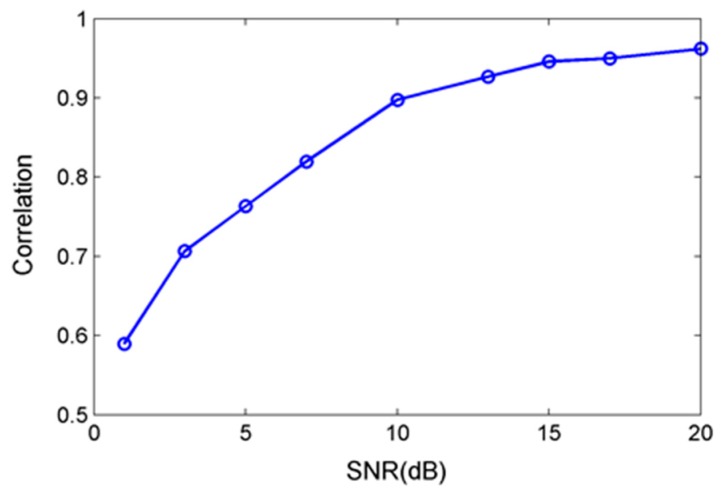
Correlation coefficients between original signal and reconstructed signal with different SNRs.

As shown in Figure 11, the reconstruction effects were good using BSBL-EM with low SNR. The reconstructed DCT coefficients and signals were similar to the original signals when the SNR is higher than 10 dB. Figure 12 shows the correlation coefficient between the original signals without noise and the reconstruction signals at different noise levels with SNR less than 20 dB.

There was a great difference between the reconstructed signal and original one under the low SNR. However, the reconstructed DCT coefficients still had similarity. The reconstruction performance was significantly improved with the increase of SNR. When the SNR exceeds 10 dB, the reconstructed signal had small difference with original ones and the correlation coefficient can reach more than 0.9.

### 4.5. Effect of Wavelet Packet Paremeters

In the past experiments, we used wavelet packet transformation matrix as the sparse representation matrix. Actually, the wavelet packet transformation matrixes have different forms, which depend on the wavelet kernels and decomposition levels. In the section, we discuss the CS reconstruction performance influenced by various wavelet packet parameters.

**Figure 13:**
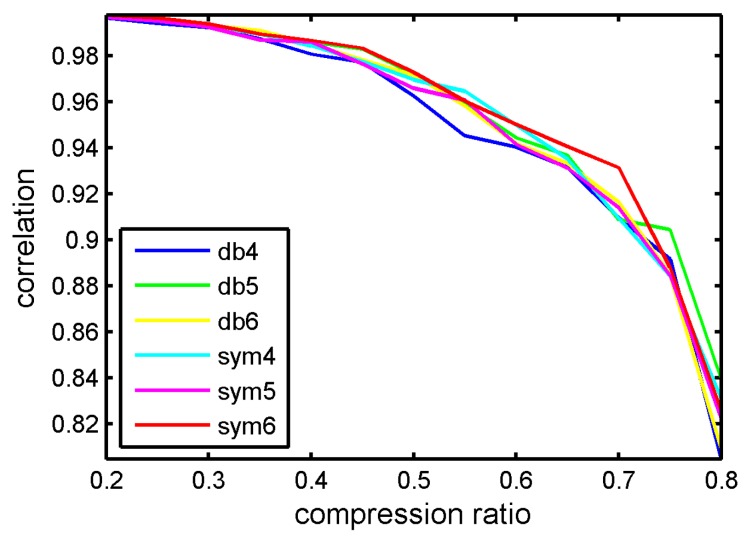
Correlation coefficients between original signal and reconstructed signal via different wavelet kernels.

The experiment data were the same as in Section 4.1. The measurement matrix was Gaussian random matrix and the reconstruction algorithm was BSBL-EM. Firstly, we set the basic wavelet types as variable. The contrastive wavelet kernels were db4, db5, db6, sym4, sym5 and sym6. The wavelet kernels were similar to the waveforms of bearing vibration signals. The correlation coefficient of reconstruction signals and original signal with different compression ratios is displayed in Figure 13.

**Figure 14:**
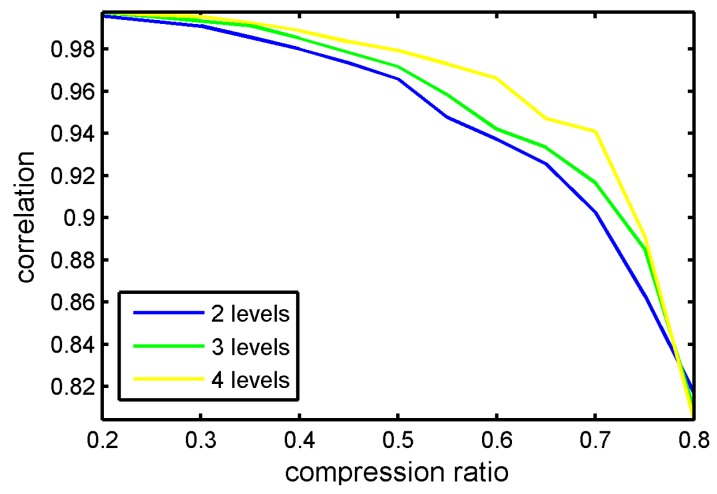
Correlation coefficients between original signal and reconstructed signal via different wavelet decomposition levels.

It can be seen in Figure 13 that various wavelets kernels have little influence to reconstruction performance. Figure 14 displays the CS reconstruction performance with different levels. In this experiment, the chosen wavelet kernel was sym6.

In Figure 14, the correlation coefficients showed slight improvements with the increase of wavelet decomposition levels; however, this change was not obvious. Therefore, it is no necessary to increase the decomposition levels to improve the performance. Actually, the reconstruction algorithms have the greatest influence on CS reconstruction performance.

### 4.6. Faults Classification

In the above sections, we only evaluated the CS performance with NMSE and correlation coefficients, which only quantitatively presented the reconstruction accuracy. The ultimate purpose of machinery condition monitoring system is identifying the bearing faults types. Will this compressed sampling and reconstruction affect the identification accuracy? In this section, we perform the faults classification using the test signals, which were reconstructed by BSBL-EM.

The faults classification method was based on feature extraction and pattern recognition. We decomposed the bearing vibration signals with sym6 wavelet packet by five levels and extracted the wavelet packet energy spectrum in 32 decomposition nodes. This wavelet packet energy spectrum was used as the feature vector. The pattern recognition method was support vector machine (SVM).

The training data were bearing vibration signals which include seven states. The states were normal, and six faults. The fault types consisted of inner-ring fault, rolling-element fault, and outer-ring fault. Each fault type was divided into two fault diameters, 7 mils and 21 mils. The number of training data totally was 1400, which consists of 200 sets of data for each bearing state. The number of testing data was totally 280, which consists of 40 sets of data for each bearing states. In each set of data, the sample points were 2000. We tested the faults classification result at different compression ratios. The measurement matrix was a Gaussian random matrix and the reconstruction algorithm was BSBL-EM. The training samples and testing sample are shown in Table 1 and Table 2, respectively.

**Figure 15:**
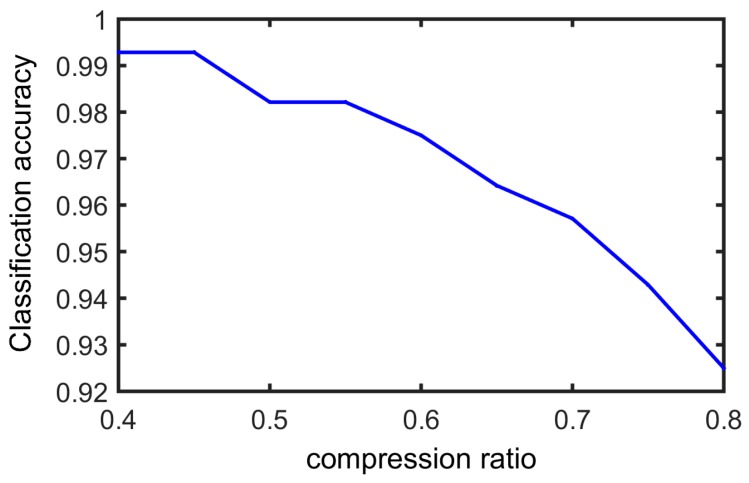
Faults classification accuracy with various CS compression ratio.

In this experiment, we test the faults classification accuracy with different CS compression ratios. Figure 15 displays the fault classification result.

It can be seen that reconstruction signals were able to preserve most of the fault information. When the compression ratio was very high, for example 80%, some information was lost and there was the lowest accuracy value; however, when the CR was lower than 70%, all types of bearing faults can be identified accurately. The successful rate of faults classification can be close to 100% when the CR is 40%.

## 5. Conclusions

This paper proposed a bearing-condition monitoring method for WSN using BSBL based on Compressed Sensing, which could reduce the burden on the WSN nodes. The compressed data acquisition in the projection space could acquire fewer measurement data with all the fault information. To accurately reconstruct the original and identify the bearing conditions, we investigated BSBL-EM as the reconstruction algorithm, which considers both the sparsity and the block property of the compressed data. The reconstruction performances of the proposed method were compared with that of several traditional CS reconstruction algorithms and some algorithms based on block sparsity. BSBL-EM demonstrated the best properties in the interpretation of the results. This paper also discussed the performance of BSBL-EM influenced by main factors. The experimental results showed the algorithm based on BSBL framework could achieve good reconstruction results under a broad range of compression ratios and various SNRs; especially, it was insensitive to block partitions. In the last section, we used SVM to identify the bearing faults. Most faults can be classified. When the CR is low, the identification accuracy is close to 100%.

## Figures and Tables

**Table 1:** Training samples.

State of the Signal	Motor Load (hp)	Motor Speed (r/min)	Number of Signals
Normal state	0	1797	50
	1	1772	50
	2	1750	50
	3	1730	50
Inner ring fault (2 diameters)	0	1797	100
	1	1772	100
	2	1750	100
	3	1730	100
Rolling element fault (2 diameters)	0	1797	100
	1	1772	100
	2	1750	100
	3	1730	100
Outer ring fault (2 diameters)	0	1797	100
	1	1772	100
	2	1750	100
	3	1730	100

**Table 2:** Testing samples.

State of the Signal	Motor Load (hp)	Motor Speed (r/min)	Number of Signals
Normal state	0	1797	10
	1	1772	10
	2	1750	10
	3	1730	10
Inner ring fault (2 diameters)	0	1797	20
	1	1772	20
	2	1750	20
	3	1730	20
Rolling element fault (2 diameters)	0	1797	20
	1	1772	20
	2	1750	20
	3	1730	20
Outer ring fault (2 diameters)	0	1797	20
	1	1772	20
	2	1750	20
	3	1730	20

## References

[1] Randall R.B., Antoni J. (2011). Rolling element bearing diagnostics-A tutorial. Mech. Syst. Signal Process..

[2] Rai A., Upadhyay S.H. (2016). A review on signal processing techniques utilized in the fault diagnosis of rolling element bearings. Tribol. Int..

[3] El-Thalji I., Jantunen E. (2015). A summary of fault modelling and predictive health monitoring of rolling element bearings. Mech. Syst. Signal Process..

[4] Patil M.S., Mathew J., RajendraKumar P.K. (2008). Bearing signature analysis as a medium for fault detection: A review. J. Tribol.-Trans. Asme.

[5] Hou L.Q., Bergmann N.W. (2012). Novel Industrial Wireless Sensor Networks for Machine Condition Monitoring and Fault Diagnosis. IEEE Trans. Instrum. Meas..

[6] Gungor V.C., Hancke G.P. (2009). Industrial Wireless Sensor Networks: Challenges, Design Principles, and Technical Approaches. IEEE Trans. Ind. Electron..

[7] Huang Q.Q., Tang B.P., Deng L. (2015). Development of high synchronous acquisition accuracy wireless sensor network for machine vibration monitoring. Measurement.

[8] Buratti C., Conti A., Dardari D., Verdone R. (2009). An Overview on Wireless Sensor Networks Technology and Evolution. Sensors.

[9] Donoho D.L. (2006). Compressed sensing. IEEE Trans. Inf. Theory.

[10] Baraniuk R.G. (2007). Compressive sensing. IEEE Signal Proc. Mag..

[11] Zhang Z.L., Jung T.P., Makeig S., Rao B.D. (2013). Compressed Sensing for Energy-Efficient Wireless Telemonitoring of Noninvasive Fetal ECG Via Block Sparse Bayesian Learning. IEEE Trans. Biomed. Eng..

[12] Zhou Z.H., Zhao J.W., Cao F.L. (2014). A novel approach for fault diagnosis of induction motor with invariant character vectors. Inf. Sci..

[13] Zhang X.P., Hu N.Q., Hu L., Chen L., Cheng Z. (2015). A bearing fault diagnosis method based on the low-dimensional compressed vibration signal. Adv. Mech. Eng..

[14] Wang Y.X., Xiang J.W., Mo Q.Y., He S.L. (2015). Compressed sparse time-frequency feature representation via compressive sensing and its applications in fault diagnosis. Measurement.

[15] Zhu K.P., Lin X., Li K.X., Jiang L.L. (2015). Compressive sensing and sparse decomposition in precision machining process monitoring: From theory to applications. Mechatronics.

[16] Tang G., Hou W., Wang H., Luo G., Ma J. (2015). Compressive Sensing of Roller Bearing Faults via Harmonic Detection from Under-Sampled Vibration Signals. Sensors.

[17] Du Z.H., Chen X.F., Zhang H., Miao H.H., Guo Y.J., Yang B.Y. (2016). Feature Identification with Compressive Measurements for Machine Fault Diagnosis. IEEE Trans. Instrum. Meas..

[18] Baraniuk R.G., Cevher V., Duarte M.F., Hegde C. (2010). Model-Based Compressive Sensing. IEEE Trans. Inf. Theory.

[19] Zhang Z.L., Rao B.D. (2013). Extension of SBL Algorithms for the Recovery of Block Sparse Signals with Intra-Block Correlation. IEEE Trans. Signal Process..

[20] Wipf D.P., Rao B.D. (2004). Sparse Bayesian learning for basis selection. IEEE Trans. Signal Process..

[21] Zhang Z.L., Jung T.P., Makeig S., Rao B.D. (2013). Compressed Sensing of EEG for Wireless Telemonitoring with Low Energy Consumption and Inexpensive Hardware. IEEE Trans. Biomed. Eng..

[22] Qaisar S., Bilal R.M., Iqbal W., Naureen M., Lee S. (2013). Compressive Sensing: From Theory to Applications, a Survey. J. Commun. Netw..

[23] Chen S., Ma S., Dl D. (2006). Atomic decomposition by basis pursuit. SIAM Rev..

[24] Tibshirani R. (1996). Regression Shrinkage and Selection via the Lasso. J. R. Stat. Soc..

[25] Mallat S.G., Zhifeng Z. (1993). Matching pursuits with time-frequency dictionaries. IEEE Trans. Signal Process..

[26] Tropp J.A., Gilbert A.C. (2007). Signal recovery from random measurements via orthogonal matching pursuit. IEEE Trans. Inf. Theory.

[27] Needell D., Vershynin R. (2009). Uniform Uncertainty Principle and Signal Recovery via Regularized Orthogonal Matching Pursuit. Found. Comput. Math..

[28] Needell D., Tropp J.A. (2010). CoSaMP: Iterative Signal Recovery from Incomplete and Inaccurate Samples. Commun. ACM.

[29] Do T.T., Gan L., Nguyen N., Tran T.D. (2008). Sparsity Adaptive Matching Pursuit Algorithm for Practical Compressed Sensing. Proceedings of the Asilomar Conference on Signals, Systems and Computers.

[30] Eldar Y.C., Kuppinger P., Bolcskei H. (2010). Block-Sparse Signals: Uncertainty Relations and Efficient Recovery. IEEE Trans. Signal Process..

[31] Baron D., Duarte M.F., Wakin M.B., Sarvotham S., Baraniuk R.G. (2009). Distributed Compressive Sensing. arXiv.

[32] Peleg T., Eldar Y.C., Elad M. (2012). Exploiting Statistical Dependencies in Sparse Representations for Signal Recovery. IEEE Trans. Signal Process..

[33] Jiang Y., Li Z.X., Zhang C., Hu C., Peng Z. (2016). On the bi-dimensional variational decomposition applied to nonstationary vibration signals for rolling bearing crack detection in coal cutters. Meas. Sci. Technol..

[34] Liu T., Yan S.Z., Zhang W. (2016). Time-frequency analysis of nonstationary vibration signals for deployable structures by using the constant-Q nonstationary gabor transform. Mech. Syst. Signal Process..

[35] Case Western Reserve University Bearing Data Center Website. http://csegroups.case.edu/bearingdatacenter.

[36] Bredies K., Lorenz D.A. (2008). Linear Convergence of Iterative Soft-Thresholding. J. Mach. Learn. Res..

[37] Efron B., Hastie T., Johnstone I., Tibshirani R. (2004). Least angle regression. Ann. Stat..

[38] Yuan M., Lin Y. (2006). Model selection and estimation in regression with grouped variables. J. R. Stat. Soc. B.

[39] Van den Berg E., Friedlander M.P. (2008). Probing the Pareto Frontier for Basis Pursuit Solutions. SIAM J. Sci. Comput..

[40] Huang J.Z., Zhang T., Metaxas D. (2011). Learning with Structured Sparsity. J. Mach. Learn. Res..

